# Effective Strategies to Recruit Young Adults Into the TXT2BFiT mHealth Randomized Controlled Trial for Weight Gain Prevention

**DOI:** 10.2196/resprot.4268

**Published:** 2015-06-05

**Authors:** Stephanie R Partridge, Kate Balestracci, Annette TY Wong, Lana Hebden, Kevin McGeechan, Elizabeth Denney-Wilson, Mark F Harris, Philayrath Phongsavan, Adrian Bauman, Margaret Allman-Farinelli

**Affiliations:** ^1^ School of Molecular Bioscience The University of Sydney Sydney Australia; ^2^ Sydney School of Public Health The University of Sydney Sydney Australia; ^3^ Faculty of Health University of Technology Sydney Sydney Australia; ^4^ Centre for Primary Health Care and Equity University of New South Wales Sydney Australia

**Keywords:** recruitment, young adults, mHealth, eHealth, weight gain prevention, external validity, cost

## Abstract

**Background:**

Younger adults are difficult to engage in preventive health, yet in Australia they are gaining more weight and increasing in waist circumference faster than middle-to-older adults. A further challenge to engaging 18- to 35-year-olds in interventions is the limited reporting of outcomes of recruitment strategies.

**Objective:**

This paper describes the outcomes of strategies used to recruit young adults to a randomized controlled trial (RCT), healthy lifestyle mHealth program, TXT2BFiT, for prevention of weight gain. The progression from enquiry through eligibility check to randomization into the trial and the costs of recruitment strategies are reported. Factors associated with nonparticipation are explored.

**Methods:**

Participants were recruited either via letters of invitation from general practitioners (GPs) or via electronic or print advertisements, including Facebook and Google—social media and advertising—university electronic newsletters, printed posters, mailbox drops, and newspapers. Participants recruited from GP invitation letters had an appointment booked with their GP for eligibility screening. Those recruited from other methods were sent an information pack to seek approval to participate from their own GP. The total number and source of enquiries were categorized according to eligibility and subsequent completion of steps to enrolment. Cost data and details of recruitment strategies were recorded.

**Results:**

From 1181 enquiries in total from all strategies, 250 (21.17%) participants were randomized. A total of 5311 invitation letters were sent from 12 GP practices—16 participating GPs. A total of 131 patients enquired with 68 participants randomized (68/74 of those eligible, 92%). The other recruitment methods yielded the remaining 182 randomized participants. Enrolment from print media was 26% of enquiries, from electronic media was 20%, and from other methods was 3%. Across all strategies the average cost of recruitment was Australian Dollar (AUD) $139 per person. The least expensive modality was electronic (AUD $37), largely due to a free feature story on one university Web home page, despite Facebook advertising costing AUD $945 per enrolment. The most expensive was print media at AUD $213 and GP letters at AUD $145 per enrolment.

**Conclusions:**

The research indicated that free electronic media was the most cost-effective strategy, with GP letters the least expensive of the paid strategies in comparison to the other strategies. This study is an important contribution for future research into efficacy, translation, and implementation of cost-effective programs for the prevention of weight gain in young adults. Procedural frameworks for recruitment protocols are required, along with systematic reporting of recruitment strategies to reduce unnecessary expenditure and allow for valuable public health prevention programs to go beyond the research setting.

**Trial Registration:**

Australian New Zealand Clinical Trials Registry (ANZCTR): ACTRN12612000924853; https://www.anzctr.org.au/Trial/Registration/TrialReview.aspx?id=362872 (Archived by WebCite at http://www.webcitation.org/6YpNfv1gI).

## Introduction

Younger adults in Australia are gaining more weight and increasing their waist circumference faster than older adults [1]. Research targeting this population of 18- to 35-year-olds has been an emerging area with several recent interventions having been completed [2-5] or underway [6-9], with demonstrated effectiveness at preventing weight gain in the short term. However, engaging young adults in public health research, particularly interventions aimed at the prevention of unhealthy weight gain, remains challenging. There is limited information reported on recruitment strategies, timelines, costs, and alterations to original recruitment protocols in response to any challenges encountered, and limited advice on the application of the recruitment techniques to community settings [10]. Current recruitment evidence is skewed toward older age groups and there is a limited representation of young adults in the literature as they are highly mobile, complicating recruitment efforts [11].

Recruitment for young adults into interventions is often timed with commencement of life events, such as starting tertiary education, moving out of the parental home, or the postpartum period. Interventions are often conducted within tertiary settings and target tertiary students [12]. This suggests that the type of young people engaging in weight gain prevention interventions may not be representative of the young adult population at large, which may reduce the external validity and translation potential [12].

Recruitment strategies reported mainly include multiple strategies, and it is not described whether the multitude of strategies is in response to low uptake from the strategy originally planned or if a combination of strategies is more effective. Traditional strategies in combination, such as posters, flyers, advertisements, email blasts, and/or information stands, are the most commonly reported [12]. In efficacy and effectiveness reporting, inadequate detailed information is provided on the recruitment materials, advertising messages, detail of location of recruitment material placement, quantity and time frame, cost of strategies, and/or the original number of people invited or making initial enquiries to participate who do not proceed to eligibility check [12].

In light of this, current research is recognizing the need for in-depth evaluation of the recruitment process for obesity prevention programs and the implications of this for translation and scalability. Research is emerging on the use of new recruitment avenues, including social media and social media advertising. New studies using Facebook advertising have been shown to be effective in recruiting young adults, particularly young women. The studies are mainly online, lifestyle, weight gain prevention programs and/or evaluations [13,14]. They show promise in recruiting a representative sample of the target population [15] and underrepresented groups [16,17].

Furthermore, little is known about eligible participants who do not engage with prevention research, and the reasons for nonparticipation [18]. There is considerable financial investment in recruiting individuals who do not complete research studies [19]. Systematic reporting of cost and effectiveness of recruitment strategies will enable researchers to select the most appropriate strategies for recruiting participants into health research studies [19]. With limited recruitment information currently reported and the large heterogeneity of studies, research interventions are not easily generalizable [20].

This paper describes the strategies used to recruit young adults to a randomized controlled trial (RCT) of an mHealth program, TXT2BFiT. The progression from enquiry through eligibility check to randomization into the trial and the costs of recruitment strategies are reported. Factors associated with nonparticipation in TXT2BFiT are explored.

## Methods

### Participants and Eligibility Criteria

The eligibility criteria for the RCT of the TXT2BFiT program included being a young adult aged 18 to 35 years [21]. Furthermore, participants had to meet the following conditions: (1) have a body mass index (BMI) of 25.0 to 31.9 kg/m^2^, or 23.0 to 24.9 kg/m^2^ with reported weight gain of more than 2 kg over the past 12 months, (2) have a fruit intake of less than two servings per day, a vegetable intake of less than five servings per day, sugar-sweetened beverage intake of at least 1 L per week, energy-dense takeout meals more than once per week, and/or engage in moderate-intensity physical activity of less than 60 minutes per day, (3) own a mobile phone capable of receiving text messages, and (4) have access to the Internet at least once a week. Exclusion criteria included (1) being pregnant or planning to fall pregnant within the next 9 months, (2) enrolled in an alternate weight loss program, (3) had lost more than 10 kg voluntarily in the past 3 months, (4) taking medications that have caused more than 2 kg of weight gain, (5) medical condition that precludes following dietary or physical recommendations, (6) history of disordered eating, and/or (7) does not speak English. The detailed eligibility and study protocol is available elsewhere [21].

### Incentives

The participant information statement informed participants that both groups would receive free advice on diet and physical activity to help them achieve and maintain a healthy weight, and that they would be compensated for their participation by receiving Australian Dollar (AUD) $10 vouchers for completing surveys and attending an in-person weigh-in (ie, a total of AUD $30 for completion of all measures).

### Recruitment

The original protocol was to enroll 354 participants, based on detecting a mean difference of 2.0 kg with *P*<.05 and 80% power, that assumed the standard deviation was 10 kg and the correlation between baseline and final weight was .8. A total of 284 participants were required—142 per arm—and accounting for a 20% dropout rate, an additional 70 participants would be needed. Two phases of recruitment were employed and are detailed below.

### Recruitment Phase 1: General Practitioner Letters

The first phase of recruitment involved personal letter invitations (see Multimedia Appendix 1) to young adult patients of participating general practitioners (GPs) recruited from Medicare Locals within the Greater Sydney Area. In July 2011, Australian primary health care services were restructured into independent entities called Medicare Locals, which are responsible for coordinating primary health care over a specified geographic area. GPs can only be recruited for study participation through the assistance of Medicare Locals.

**Figure 1:**
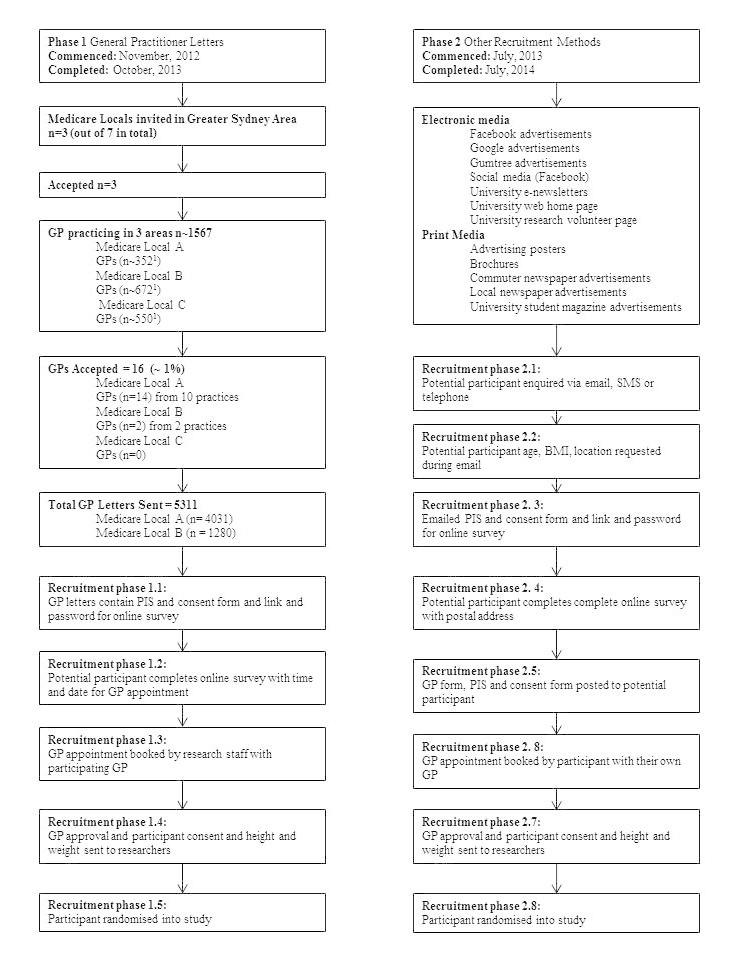
Flow diagram for recruitment phases of the TXT2BFiT study. The number of GPs in each area is approximate. Information obtained from Medicare Local website [24-26].

Recruitment Phase 1 commenced in November 2012 (see Figure 1) with three of the seven Medicare Locals in the Greater Sydney Area invited and willing to participate. Our original research and calculations indicated that 3% of patients in a GP practice would be eligible, which meant there would be 60 eligible patients in an average practice. Based on previous research [22,23], if 25% of patients accepted an invitation, it meant that 24 practices would be needed and recruitment was expected to last for 18 months. From these Medicare Locals, 16 GPs from 12 practices—14 (14/352, 4.0%) from Medicare Local A, 2 (2/672, 0.3%) from Medicare Local B, and 0 (0%) from Medicare Local C—agreed to join the study using the latest available GP numbers for each area [24-26]. A total of 5311 letters of invitation were sent to young adult patients. GPs do not routinely collect anthropometric data, including weight and height, and therefore all young adults in the required age range were eligible to receive a letter of invitation, regardless of BMI.

### Screening for Eligibility: Phase 1, General Practitioner Letters

The invitation letter directed prospective participants to an online survey to screen for eligibility. Questions in the screening survey were structured such that ineligible participants were redirected to a national social marketing website for healthy eating and physical activity promotion [27,28]. Eligible participants reaching the end of the survey were able to nominate dates and times to attend an appointment with the GP—paid for by the study—who had invited them via the letter (see Figure 1 and Multimedia Appendix 1). This 10-minute appointment was booked on the participant’s behalf and details sent to the patient by research staff in a confirmatory short message service (SMS) text message. At the appointment, the GP measured the participant’s weight (kg) and height (cm), approved their participation, and collected the participant’s signed, written informed consent to enter into the trial. Signed consent forms were returned to the researchers with the participants' anthropometric data.

### Recruitment Phase 2: Other Forms of Recruitment

Phase 2 of recruitment ran from July 2013 (see Figure 1) until July 2014, involving two main avenues of electronic and print media (see Table 1) because recruitment from Phase 1 slowed. This range of other recruitment strategies and materials using a variety of modalities is described below (see Table 1). Advertising messages were brief, using simple language. Furthermore, advertisements were accompanied by a TXT2BFiT logo, along with healthy and/or unhealthy food images, and/or positive physical activity images (see Multimedia Appendix 1).

### Electronic Media Recruitment

Electronic media utilized Facebook and Gumtree advertisements, social media through the use of a TXT2BFiT Facebook page, university e-newsletter, university Web home page news story, and a consistent listing on two university research volunteer pages for the duration of recruitment (see Table 1). Paid Facebook and Google advertising was used three and four times, respectively. To generate these advertisements, the target population was defined, along with a specific budget and time frame. After the duration of the advertisement listing, advertising data were downloaded and interpreted. Free advertising on the Gumtree website—a network of free online classifieds and community websites—was updated 16 times and included two different low-cost advertisements. The TXT2BFiT Facebook page status was updated weekly for the duration of the recruitment period. Anyone interested "requested to be a friend" of the page. Friends were predominantly those of the research staff and their friends and family. Once saturation was reached (ie, no new research staff members to share the page with their "friends"), this avenue provided no further enquiries. The research study was featured in the e-newsletters of two universities, which were sent to all enrolled undergraduate and postgraduate students and staff, and was the topic of a feature story on the Web home page of one university for approximately one week. For the duration of recruitment, the study was listed on two separate universities' research volunteer Web pages and briefly mentioned in lectures at a third university.

### Print Media Recruitment

Print media consisted of advertising posters, brochures, commuter and local newspaper advertisements, and university student magazines. Over the 12-month time period (excluding semester breaks), posters were displayed on two university campuses at popular locations and replaced weekly (see Table 1). Posters were also displayed at various community locations and at Technical and Further Education (TAFE) institutions at the beginning of two semesters. Brochures were mostly delivered by research staff and students (n=35,002), but 20,000 brochures were delivered by a distribution company. Suburbs within the Greater Sydney Area with a high percentage of young adults based on census data were targeted. Newspaper and magazine paid advertising was conducted in five local district newspapers and magazines and one newspaper, widely distributed to commuters for free at train stations, on six occasions.

**Table 1:** TXT2BFiT recruitment strategy descriptions.

Modality	Recruitment strategy	Recruitment strategy detail
GP^a^	GP letter	5311 letters sent from 12 participating practices (16 participating GPs) Personally addressed letter with GP letterhead, TXT2BFiT and university logo, and instructions provided on how to access screener survey AUD^b^ $500 GP fee per practice paid for time spent to access to their database for potential participants Additional AUD $3827.22 for postage and printing
**Electronic media**		
	Facebook advertisement	Paid advertisements over 22 days Link with image accompanying website (Multimedia Appendix 1) on the right-hand advertising column of Facebook—targeted to location and age demographics See reach data in Results, Effectiveness and Cost of Different Recruitment Strategies section
	Google advertisement	Paid advertisements over 7 days Top-of-page and side-advertisement text See reach data in Results, Effectiveness and Cost of Different Recruitment Strategies section
	Gumtree advertisement	16 free advertisements Two paid advertisements
	Social media (Facebook)	TXT2BFiT Facebook page Weekly updates for 12 months Status update examples accompanied by a photo (Multimedia Appendix 1)
	University e-newsletter	Three featured newsletters sent to all students at three universities
	University Web home page	One banner news story on the home page of one university
	University research volunteer Web page	Listed for the duration of the study recruitment (21 months)
**Print media**		
	Advertising poster	Placed at poster locations at two university campuses during semester (14 months), including a brief advertisement at the end of PowerPoint lectures at one university Posters placed at various community locations Posters placed at TAFE^c^ institutes at the beginning of two semesters
	Brochures	54,872 delivered in total 19,870 company delivered and 35,002 delivered by research staff
	Commuter newspaper advertisement	Six advertisements Handed out during peak hours, 2:30-7:00 PM, on weekdays for train commuters at CBD^d^ locations 663,000 readers, predominantly 18-39 years, in three major capitals in Australia (only advertised in Sydney)^e^ and 73.5% of readers live outside inner-city Sydney
	Local newspaper advertisement	Two advertisements in two different local newspapers
	University students' magazines	Two advertisements in two different university magazines
Word of mouth	Friend/family	Heard about the study through friends or family
Other	Unknown	Participant did not respond to email and/or could not recall where they heard about the study

^a^General practitioner (GP).

^b^Australian Dollar (AUD).

^c^Technical and Further Education (TAFE).

^d^Central business district (CBD).

^e^Obtained from the mX website [29].

### Screening for Eligibility: Phase 2, Other Forms of Recruitment

Potential participants registered their interest via email or SMS text message. Researchers screened participants to assess if their age, BMI, and the location of their GP made them eligible. Subjects reported how they heard about the research study (ie, recruitment source). Ineligible participants exited the survey and were redirected to national social marketing sites as detailed above for Phase 1 participants. Eligible participants reaching the end of the survey provided their postal address and received a pack containing a letter explaining the study to the participant, a consent form, a participant information sheet, and a letter explaining the study to the GP with an approval form for the GP to sign as detailed above. However, in this case the participant booked their own appointment, which was paid for by the study (see Figure 1).

### Data Collection Procedures

All participant enquiries were recorded in a database. The online survey website, SurveyMonkey [30], collected data on demographics, including gender, postcode—for categorizing socioeconomic data [31]—recruitment modality and strategy, and the eligibility criteria. Detailed data were also collected on the number of GPs in each Medicare Local [24-26]; number of participating GPs; number of GP letters sent and the associated cost; paid advertising costs; number, location, and time frame of brochure deliveries; and time frame of advertising poster distribution and social media updates. All data were recorded in a database.

### Statistical Analysis

Descriptive statistics for continuous measures, including counts and percentages for total number of enquiries, total eligible participants, and total participants randomized, are provided for each recruitment modality and strategy. Total costs (AUD $) are reported per recruitment modality and strategy, with the average cost calculated per participant randomized and per eligible participant. Results from each recruitment method are discussed in comparison to each other, as there were not standards or targets defined in the literature for recruitment methodology.

Logistic regression was used to assess any differences in baseline characteristics between eligible participants who were or were not randomized into the study. Characteristics included gender, BMI, postcode—for categorizing socioeconomic data [31]—and recruitment modality and strategy.

### Ethics

Materials and methods of the TXT2BFiT RCT were approved by the University of Sydney Human Research Ethics Committee in September 2012 (Approval Number 15226). The trial is registered with the Australian New Zealand Clinical Trials Registry (ACTRN 12612000924853).

## Results

### Flow of Participants From Recruitment to Randomization

**Figure 2:**
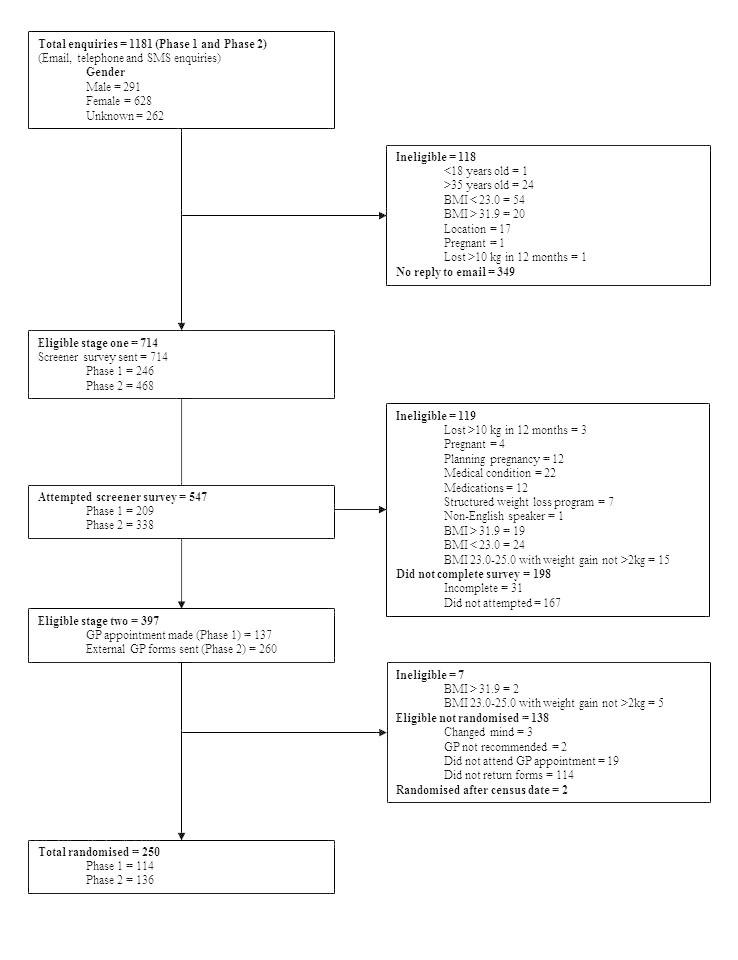
Flow diagram for recruitment of participants into the TXT2BFiT study.

A total of 1181 people enquired from Phase 1 and 2—24.64% (291/1181) male, 53.18% (628/1181) female, and 22.18% (262/1181) remaining unknown (see Figure 2). Of the 1181 people, 349 (29.55%) did not enquire further from their initial enquiry and 118 (9.99%) people were ineligible primarily due to their BMI being below 23 kg/m^2^. The remaining 714 (60.46%) were sent links to the screener surveys as detailed above. Of the 714 remaining participants, 119 (16.7%) were ineligible on completion of the screener survey and a further 198 (27.7%) people did not complete it—167 of the 198 (84.3%) did not even attempt the survey. GP appointments were made for 137 people from phase 1, of whom 13.9% (19/137) did not attend. GP information packs were sent to 260 people from Phase 2, of whom nearly half (113/260, 43.5%) did not see a GP to complete screening. A total of 250 out of 1181 (21.17%) participants were randomized, with over half resulting from recruitment methods other than GP letters (see Table 2).

**Table 2:** Total enquiries, eligible and randomized, and cost (AUD $) per recruitment strategy for the TXT2BFiT study.

Modality	Recruitment strategy	Total enquiries, n (%)	Total eligible, n (% per enquiry)	Total randomized, n (% per eligible)	Total cost^a^, AUD^b^ $	Cost per participant randomized^c^, AUD $
All modalities	All recruitment methods	1181 (100)	390 (33.02)	250 (64.1)	34,638.96	138.56
General practice	General practice letter	131 (11.09)	74 (56.5)	68 (92)	9827.22	144.52
**Electronic media**						
	All electronic media	335 (28.37)	118 (35.2)	68 (57.6)	2498.06	36.74
	Facebook advertisement	13 (1.10)	10 (77)	2 (20)	1890.66	945.33
	Google advertisement	4 (0.34)	3 (75)	1 (33)	571.45	571.45
	Gumtree advertisement	50 (4.23)	10 (20)	3 (30)	35.95	11.98
	Social media (Facebook)	7 (0.59)	7 (100)	3 (43)	No cost	N/A^d^
	University e-newsletter	76 (6.44)	43 (57)	23 (53)	No cost	N/A
	University Web home page	164 (13.89)	35 (21.3)	28 (80)	No cost	N/A
	University research volunteer page	21 (1.78)	10 (48)	8 (80)	No cost	N/A
**Print media**						
	All print media	410 (34.72)	180 (43.9)	105 (58.3)	22,313.68	212.51
	Advertising poster	109 (9.23)	48 (44.0)	29 (60)	No cost	N/A
	Brochures	135 (11.43)	67 (49.6)	41 (61)	13,631.01	332.46
	Commuter newspaper advertisements	163 (13.80)	63 (38.7)	34 (54)	6875.00	202.21
	Local newspaper advertisements	0 (0)	0 (0)	0 (0)	1067.67	No one randomized
	University students' magazines	3 (0.25)	2 (67)	1 (50)	740.00	740.00
Word of mouth	Friend/family	30 (2.54)	8 (27)	5 (63)	No cost	N/A
Other	Unknown	275 (23.29)	10 (3.6)	4 (40)	N/A	N/A

^a^Research staff costs were not included.

^b^Australian Dollar (AUD).

^c^All randomized participants had a AUD $55 general practitioner visit paid for that was not included in this analysis.

^d^Not applicable (N/A).

### Effectiveness and Cost of Different Recruitment Strategies

Table 2 shows the number of enquiries and eligible participants, the number of subjects enrolled and randomized, and their respective costs stratified by modality type and the strategy subcategories. Letters sent by GPs resulted in 131 enquiries out of a total of 1181 (11.09%), with 74 eligible out of 131 (56.5%). Electronic media resulted in 335 enquiries out of a total of 1181 (28.37%), with 118 eligible out of 335 (35.2%). Print media achieved 410 enquiries out of a total of 1181 (34.72%), with 180 eligible out of 410 (43.9%). The conversion of eligible enquiries into enrolments and randomization indicates that GPs (68/74 eligible per strategy, 92%) were highest, followed by print media (68/118 eligible per strategy, 57.6%) and electronic media (105/180 eligible, 58.3%). A total of AUD $34,638.96 was spent, which meant an average of AUD $138.56 was spent per participant enrolled and randomized. This was AUD $144.52 for GPs, AUD $36.74 for electronic media, and AUD $221.51 for print media. When the substrategies within the major modalities were examined, it was found that the university newsletters yielded the second-most enquiries with more than half eligible, and just over half of these enrolled and randomized, and there was no direct cost. For print media, the brochures gave the second-most enquiries with most eligibility and highest enrolment, but the cost was high at AUD $332.46 per person.

Electronic media had a wide range of costs per strategy—AUD $0 to AUD $944 per participant randomized. Targeted paid advertising on Facebook reached 953,007 people (see Table 3 and Table 4), yet only attracted 13 enquiries and made this strategy the most expensive, costing AUD $945 per participant randomized. Likewise, Google advertising was served to 605,504 people over 7 days (see Table 4 and Table 5), at a cost of AUD $571 with four enquiries and one participant randomized.

Print media was the most expensive modality per participant randomized, at AUD $213 (see Table 2) and was the most time-consuming for research staff (ie, brochure distribution and poster placement). It resulted in the most enquiries (410/1181, 34.72%) and provided the greatest proportion of participants randomized (105/250, 42.0%). All print media strategies had similar enrolment rates from eligible participants, despite the advertising materials varying considerably in the information provided (see Multimedia Appendix 1). There were no enquires from paid company-delivered brochures (see Table 1). Brochures delivered individually by the research staff resulted in 136 enquiries. Word-of-mouth (ie, family/friend) recruitment only accounted for 2.0% (5/250) of the total participants randomized.

**Table 3:** Facebook TXT2BFiT advertisement data for website clicks on right-hand column advertisements on desktop computers over 4 days in 2013 and over 18 days in 2014^a^.

Advertisement data	Year and duration						
	2013						2014
	2 days, n, %, or AUD^b^$		1 day, n, %, or AUD $		1 day, n, %, or AUD $		18 days, n, %, or AUD $
Reach, n	72,979	119,661	42,310	79,731	48,525	86,086	503,715
Frequency^c^, n	3.16	4.39	2.96	4.28	3.27	4.91	10
Impressions, n	230,500	525,364	125,127	341,109	158,684	422,880	5,196,868
Clicks, n	46	143	34	93	28	108	1061
Unique clicks, n	45	132	34	91	27	105	0
CTR^d^, %	0.02	0.03	0.03	0.03	0.02	0.03	0.02
uCTR^e^, %	0.06	0.11	0.08	0.11	0.06	0.12	0.21
Spent, AUD $	51.68	148.32	27.03	72.97	21.89	78.11	1490.70
CPM^f^_,_AUD $	0.22	0.28	0.22	0.21	0.14	0.18	0.29
Cost per 1000 people reached, AUD $	0.71	1.24	0.64	0.92	0.45	0.91	N/A^g^
CPC^h^, AUD $	1.12	1.04	0.80	0.78	0.78	0.72	1.40
Cost per unique click, AUD $	1.15	1.12	0.80	0.80	0.81	0.74	N/A
Actions, n	47	144	35	93	29	111	N/A
People taking action, n	72,979	119,661	42,310	79,731	48,525	86,086	N/A

^a^Downloaded from Facebook Ads Reporting.

^b^Australian Dollar (AUD).

^c^Frequency is the average number of times the advertisement was served to each person.

^d^Click-through rate (CTR).

^e^Unique click-through rate (uCTR).

^f^Cost per 1000 impressions (CPM).

^g^Not applicable (N/A).

^h^Cost per click (CPC).

**Table 4:** Facebook and Google TXT2BFiT advertisement data definitions.

Term	Definition^a^
Campaign, placement	A group of advertisement sets that share the same objective, where the advertisement was served on Facebook and Google
Duration	Length of the advertising
Reach	The number of people the advertisement was served to
Frequency	The average number of times the advertisement was served to each person
Impressions	The number of times the advertising was served. On mobile apps, an advertisement is counted as served the first time it is viewed. On other Facebook interfaces, an advertisement is served the first time it is placed in a person's News Feed or each time it is placed in the right-hand column.
Clicks	The total number of clicks on the advertisement. Depending on what is being promoted, this can include page likes, event responses, or app installs.
Unique clicks	The total number of unique people who have clicked on the advertisement. For example, if 3 people click on the same advertisement 5 times, it will count as 3 unique clicks.
CTR^b^	The number of clicks received divided by the number of impressions
uCTR^c^	The number of people who clicked on the advertisement divided by the number of people you reached. For example, if you received 20 unique clicks and your advertisement was served to 1000 unique people, your unique click-through rate would be 2%.
Spent/cost	The total amount spent so far
CPM^d^	The average cost paid to have 1000 impressions on the advertisement
Cost per 1000 people reached	The average amount paid to have the advertisement served to 1000 unique people
CPC^e^	The average cost per click for the advertisements, calculated as the amount spent divided by the number of clicks received
Cost per unique click	The average cost per unique click for the advertisements, calculated as the amount spent divided by the number of unique clicks received
Actions	The number of actions taken on the advertisement—page, app, or event—after the advertisement was served to someone, even if they didn’t click on it. Actions include page likes, app installs, conversions, event responses, and more. For example, 2 page likes and 2 comments would be counted as 4 actions.
People taking action	The number of unique people who took action such as liking the page or installing the app as a result of the advertisement. For example, if the same person likes and comments on a post, they will be counted as 1 unique person.
Average position	Average position of where the advertisement ranks compared to other ads

^a^Definitions available from Facebook Ads Reporting and Google Ads Reporting.

^b^Click-through rate (CTR).

^c^Unique click-through rate (uCTR).

^d^Cost per 1000 impressions (CPM).

^e^Cost per click (CPC).

**Table 5:** Google TXT2BFiT advertisement data for top-of-page and side-advertisement text over 7 days in 2013^a^.

Advertisement data	Year 2013
Duration, days	7
Clicks, n	601
Impressions, n	605,054
CTR^b^, %	0.10
CPC^c^, AUD^d^$	0.97
Cost, AUD $	581.95
Average position, rank	2.3

^a^Downloaded from Google Ads Reporting.

^b^Click-through rate (CTR).

^c^Cost per click (CPC).

^d^Australian Dollar (AUD).

### Eligible Nonrandomized Participants Versus Randomized Participants

During recruitment, 138 potential participants were identified that were not randomized into the study. Reasons included failure to return their consent form, nonattendance at a GP appointment booked on their behalf, changing their mind, and/or their GP did not recommend the study (not for medical reasons) (see Figure 2). Logistic regression models demonstrated that females were less likely to go on to randomization compared to males—odds ratio (OR) 0.64 (95% CI 0.41-1.00) (see Table 6). Eligible participants recruited through a GP letter were more likely to be randomized than those recruited through all other recruitment modalities—OR 1.8 (95% CI 1.4-2.4) (see Table 6).

**Table 6:** Baseline health characteristics, recruitment modalities, and strategies of eligible participants who did not participate in the TXT2BFiT study (n=138) versus randomized participants (n=250)^a^.

Characteristic, modality, or strategy		Total eligible not randomized, n (%)	Total randomized, n (%)
**Gender**			
	Male	40 (29.0)	97 (38.8)
	Female	98 (71.0)	153 (61.2)
**SES quintiles** ^b^			
	0-60%^c^	10 (7.2)	15 (6.0)
	61-80%	29 (21.0)	45 (18.0)
	81-100% (highest)	99 (71.7)	189 (75.6)
**Recruitment modality**			
	GP^d^ letter	6 (4.3)	68 (27.2)
	Electronic media	50 (36.2)	69 (27.6)
	Print media	73 (52.9)	104 (41.6)
	Other	9 (6.5)	9 (3.6)
**Recruitment strategy**			
	GP letter	6 (4.3)	68 (27.2)
	Facebook advertisement	8 (5.8)	2 (0.8)
	Google advertisement	2 (1.4)	1 (0.4)
	Gumtree advertisement	7 (5.1)	3 (1.2)
	Social media (Facebook)	4 (2.9)	3 (1.2)
	University e-newsletter	20 (14.5)	23 (9.2)
	University Web home page	7 (5.1)	28 (11.2)
	University research volunteer page	2 (1.4)	8 (3.2)
	Advertising poster	18 (13.0)	29 (11.6)
	Brochures	25 (18.1)	41 (16.4)
	Commuter newspaper advertisements	29 (21.0)	34 (13.6)
	Local newspaper advertisements	0 (0)	0 (0)
	University students' magazines	1 (0.7)	1 (0.4)
	Word of mouth	3 (2.2)	5 (2.0)
	Unknown	6 (4.3)	4 (1.6)
**BMI** ^e^ **, kg/m** ^**2**^			
	23.0-24.9	31 (22.5)	58 (23.2)
	25.0-29.9	87 (63.0)	156 (62.4)
	30.0-32.0	20 (14.5)	36 (14.4)

^a^All data obtained from screener survey.

^b^Socioeconomic status (SES) by population percentile for Socio-Economic Indexes for Areas (SEIFA) Index of Relative Socio-economic Advantage and Disadvantage (IRSAD) (Australian Bureau of Statistics, 2008).

^c^Combined bottom three quintiles.

^d^General practioner (GP).

^e^Body mass index (BMI).

## Discussion

### Principal Findings

The TXT2BFiT mHealth study, aimed at preventing weight gain in 18- to 35-year-olds, recruited 250 participants over an 18-month time period, with 21% of those expressing interest randomized into the study. The recruitment protocol originally planned to enroll 354 participants from GP letters (Phase 1) but the inability of two Medicare Locals to fully engage potential participants, and lower than expected response from patients, lead to a second recruitment phase using other means. Free or low-cost electronic media appeared to be the most cost-effective and time-efficient strategy to recruit young adults. However, electronic strategies that had a greater reach (ie, Facebook and Google advertising) achieved low numbers of enquiries. GP letters were a more effective recruitment strategy than print media in terms of cost and eligibility from enquiries. Both GPs and paid print media strategies, including brochures and commuter newspaper advertisements, potentially reached a more diverse population. Men were more likely than women to follow through with enrolment into the study.

Recruitment into a face-to-face, group weight gain prevention intervention in the United States for 18- to 35-year-olds, with a BMI between 21 and 30 kg/m^2^ and similar recruitment time frame (19 months), reported 10% of total enquiries were randomized, costing US $233 per participant randomized, which excluded research staff costs [32]. In Australia, a face-to-face individual weight management study recruited 50 overweight or obese (BMI ≥ 27.5 kg/m^2^) young women 18 to 25 years over a 2-year period, and cost AUD $308 per person randomized, however, this included research staff costs. If staff costs were removed, this would be reduced to AUD $62 per person randomized [33]. In this study, one full-time research staff member was employed at a cost of AUD $100,000 per annum, but it is estimated that no more than 30% of the time over the 18 months was spent on recruitment, as they also were involved with intervention delivery. In addition, a research student spent 1 day per week on recruitment. Taking this into account would mean recruitment per participant would be estimated at AUD $319. This mHealth study utilized low-cost recruitment strategies with no budget for mass media. Recruitment for young adults to the previously mentioned weight gain prevention program had limited success with mass media television advertising, costing over US $1000 per person randomized, and having a low percentage of the total randomized [32]. However, mass media campaigns have been shown to be an effective method of promoting a telephone-based, state-wide lifestyle program, particularly targeting socioeconomically disadvantaged and overweight participants [34], although no cost data were presented for this program and results included a wide age range of participants. Process evaluation indicated that when developing mass media communications, preference should be given to specifically designed and tailored messaging that explains, models, and displays the relevant contact details for as long as possible to facilitate contact to the program [35]. Secondary referral recruitment, such as GP referral, was recommended as a supplement to the mass media campaigns.

Using Medicare Locals to invite GPs to join in with participant recruitment was included as a feasible method for recruitment in this study due to the reported success in other prevention interventions, although they focused on older adults with existing metabolic risk factors [22]. Targeted recruitment and high enquiry rate (30.6%) was possible in older age groups as anthropometric and metabolic risk factors were documented in a patient’s medical records [23]. A fee of AUD $500 per practice for study participants was considered a worthwhile investment by researchers. Young adults had a lower-than-expected enquiry rate to the GP letters (2.5%), with approximately 5.7 participants per practice randomized (range 0 to 18). The study would have required an additional 50 practices—50% greater than anticipated, 62 in total—at the rate observed, costing over AUD $50,000 to randomize the original target of 354 participants. Weight and height are rarely recorded for young adult patients in general practice, and this limited the targeting of invitation letters to patients at risk of weight gain. The transient nature of young adults may suggest that having a long-term GP is less likely, and a proportion of the GP letters may not have been reached by the recipient. For the young adults deemed eligible to participate in Phase 2 of recruitment, the required paid visit to the GP was for ethical reasons, however, this may have been a barrier to enrolment into the RCT.

GPs also showed low interest in engaging young adults into the study, despite being compensated AUD $500 for allowing access to their patient database. Reasons for lack of interest require further research. Primary health care (ie, Medicare Locals) were undergoing extensive restructuring which negatively impacted on cooperation of the Medicare Locals and participation by GPs. Only one of three Medicare Locals recruited the anticipated number of GPs—eight per Local—and one Medicare Local took 12 months to be sufficiently organized to participate and then failed to recruit any GPs. The Medicare Local network has yet again been dissembled with a change of government. This avenue appears to have some degree of instability and may have hindered recruitment efforts. In addition, there is some evidence to suggest GPs believe lifestyle interventions are ineffective [36]. No published RCT aiming to prevent weight gain in young adults utilized the general practice setting to recruit participants. Recruitment strategies are generally poorly reported as we have previously published [12]. Brief descriptions of recruitment methods were reported in only 62% of studies [12], which included using existing databases, mailings, posters, flyers, advertisements, email blasts, and information stands. The effectiveness and cost of traditional strategies in this population have only been reported in two interventions from Western countries [32,33] and other populations at risk of weight gain, such as young families [37]. Formative research into recruitment strategies is providing valuable evidence that may assist in efficient and systematic recruitment processes [32,38].

Among the possible reasons why enquiries were low, focus groups of overweight young adults show health, social image, and self-confidence were reasons identified for pursuing weight loss [38]. However, young adult men, particularly those with reported weight gain and with an overweight BMI (≥ 25.0 kg/m^2^), reported needing to gain greater than 6 kg before becoming concerned [39]. This is consistent with Australian data showing that overweight men recognize a growing societal concern with many health-related implications with weight gain, but do not feel this was something that affected them personally at their current life stage [40]. It is unclear whether identifying poor behavioral choices associated with weight gain, such as inadequate fruit and vegetable consumption, high intake of sugary soft drinks, increased frequency of takeout meals, and low levels of physical activity, were reasons for young adults to engage in prevention interventions. Thus, advertising for the TXT2BFiT study used images focused on weight gain, depicting an overweight man with central adiposity, as well as scales. Formative research into advertising materials suggested young adults would avoid advertising focusing on images of scales [32], but this was not published at the time TXT2BFiT materials were designed. Now our slogan “gained a few kilos” may be more powerful if the cumulative effect of excess weight gain over time was advertised.

Young adults reported they would be unlikely to click on paid adverting for recruitment to a weight gain prevention program on social networking sites such as Facebook [32]. Facebook paid advertising proved ineffective in the current study, costing approximately seven times the average cost per participant randomized, and had a low enquiry rate despite the high reach. Australian research that has demonstrated recruitment through free advertising on a university Facebook page has been shown to be effective for recruiting young women, 18 to 30 years, to an online weight management survey [13], and was comparable to the e-newsletter strategy used in this study. However, this may limit the representativeness of the sample as the target audience is restricted to the university population. Facebook paid advertising has been shown to be a cost-effective recruitment strategy—US $20 per compliant participant—for online health surveys in 18- to 25-year-old women [15]. Moreover, this has been shown to reach a more representative sample of the population, with success in recruiting nonurban and low-income women [15,16]. Facebook paid advertising was compared to social networking and social marketing for parents of adolescent children and was shown to recruit nearly three times as many participants in less time and at less cost—204 participants over 2 months at AUD $5.94 per participant versus 74 participants over 8 months at AUD $58.70 per participant, respectively [14]. Traditional survey methods for young women such as mailings are becoming costly, with a recent study reporting a three-fold increase—from AUD $30 to just over AUD $100—in the cost to recruit young women aged 18 to 23 years for a national Australian survey [41].

Recent CONSORT (2010) guidelines recommend clearly displaying the flow of participants throughout a study and that studies report the number of eligible participants prior to randomization, yet they do not insist on the need to report the original overall number of responders invited to participate (prior to eligibility) [42]. Despite identifying recruitment as part of their framework, the CONSORT guidelines do not define the actions needed to identify and recruit potential populations of participants. There is an absence of conceptual frameworks for recruitment to intervention studies and also a lack of procedural models. There is a need to identify what factors are effective in engaging eligible participants to improve the external validity of the research study and to establish recruitment goals based on the target population to engage with population subgroups.

### Limitations

The cost data reported from this research study is limited to cost per strategy and does not include research staff time. Time spent on recruitment was difficult to calculate due to research staff having multiple roles within the research study. This study had one full-time staff member or less employed at any one time. This needs to be accounted for with future cost analysis. For future translation potential, the recruitment process for this study has areas where improvements can be implemented to attract only eligible participants. No formative research was conducted to inform the development of the recruitment materials nor any focus group discussions of the advertisements employed. In future, it is recommended that formative research be conducted prior to scale-up. The recruitment materials can contain a quick response code for mobile phones, which can provide the potential participant with an instant and direct link to the program website explaining eligibility, which will eliminate the need for prior email correspondence. Self-reported measurements have been shown to accurately identify overweight and/or obesity in young people [43]. The requirement for a GP visit resulted in a large dropout of eligible participants prior to attending an appointment. The visit may have been a potential barrier for participants, and the necessity of this step will be investigated further before translation and scale-up in the wider community. Reasons for eligible participants not participating in research studies requires further exploration. Considerable cost is invested in recruiting participants who drop out and researching the reasons for nonparticipation may lead to future cost saving in population obesity prevention programs. Finally, the recruitment strategies resulted in a sample skewed toward a higher SES advantage, but evidence is lacking and this requires further investigation. Targeted recruitment for socially disadvantaged and minority groups needs to be established for future effectiveness research.

### Conclusions

This study is an important contribution for future research into efficacy, translation, and implementation of cost-effective programs for the prevention of weight gain in young adults in general, and in using eHealth. The research indicated that free electronic media was the most cost-effective strategy, with GP letters the most effective of the paid strategies. The results provide guidance for future research, as currently there is limited published research available on the cost and effectiveness of recruitment strategies. The large heterogeneity between published studies shows conflicting information on the best strategies to engage young adults. Procedural frameworks for recruitment protocols are required, along with systematic reporting of recruitment strategies to reduce unnecessary expenditure and allow for valuable public health prevention programs to go beyond the research setting.

## Appendix

Multimedia Appendix 1 Examples of the recruitment materials for each modality.

## Abbreviations

AUD: Australian Dollar
BMI: body mass index
CBD: central business district
CPC: cost per click
CPM: cost per 1000 impressions
CTR: click-through rate
GP: general practitioner
HCF: Hospitals Contribution Fund
IRSAD: Index of Relative Socio-economic Advantage and Disadvantage
OR: odds ratio
RCT: randomized controlled trial
SEIFA: Socio-Economic Indexes for Areas
SES: socioeconomic status
SMS: short message service
TAFE: Technical and Further Education
uCTR: unique click-through rate
