# Supplementary material for: Effective Strategies to Recruit Young Adults Into the TXT2BFiT mHealth Randomized Controlled Trial for Weight Gain Prevention
Source: JMIR Res Protoc. 2015 Jun 5;4(2):e66. doi: 10.2196/resprot.4268 (PMC4526902; doi:10.2196/resprot.4268)
Supplement: Multimedia Appendix 1 [file resprot_v4i2e66_app1.pdf]

Multimedia Appendix 1

[Patient Full Name] DATE

[Patient Full Street Address]

[Patient suburb, NSW, zip code]

Dear [Patient First (Given) Name],

Your Doctor's Medical Practice is taking part in a study called **'TXT2BFiT - A Healthy Lifestyle Program for Young Adults'**. This letter is to invite you to participate in the **TXT2BFiT** study program. Attached with this letter is a *Participant Information Statement* which provides you more detail of the study, as well as a *Consent Form* if you choose to participate.

What is the TXT2BFiT study about?

Gaining weight (body fat) is common during young adulthood. This is because we like to think we are healthy when young, so maintaining a healthy lifestyle often comes last behind work, study, social commitments and other life events. To help you improve your eating and exercise habits and avoid this weight gain, health professionals (Accredited Dietitians and Physical Activity specialist) at the University of Sydney want to test their **TXT2BFiT** program to see how helpful it is before offering it to other young adults.

How do I participate?

We are looking for young adults aged 18 to 35 (inclusive) who are in the slightly to moderately overweight range. If you feel this study is right for you, please read through the *Participant Information Statement* included with this letter. If you then decide you would like to participate, please complete our Screener Survey here: **http://txt2bfit.com/survey**

To log in to the survey, after clicking on the 'continue to survey' button, please type in the following password for security purposes:

icanbfit

Completing this survey is completely voluntary. You are not under any obligation to complete it. Any information you provide in the survey will be kept strictly confidential by the researchers. You can stop the survey at any time. When you click 'done' at the end of the survey you are consenting to providing us with this information. However, it does not mean you consent to participating in the TXT2BFiT study program.

Please complete the whole survey in one go. Please do NOT close your web browser during the survey OR leave the survey idle, causing the browser to time out, as your responses will not be saved and you will have to start the survey again.

At the end of the survey, we will ask you what dates and times you would be available for a brief (10 minute) appointment with your doctor which is free (no cost) to you. We will then book this appointment for you and send you a confirmation SMS text message with the date and time of your booked appointment. At this appointment your doctor or practice nurse will measure your weight and height and assess whether this program is appropriate for you.

Before attending this appointment, please sign the *Consent Form* posted to you with this letter and bring this form, signed, with you to your appointment.

Please feel free to send me an email at info.txt2bfit@sydney.edu.au or call 9351 3815 if you have any questions.

Sincerely,

Figure 1 Example General Practitioner recruitment letters sent to selected patients of general practice

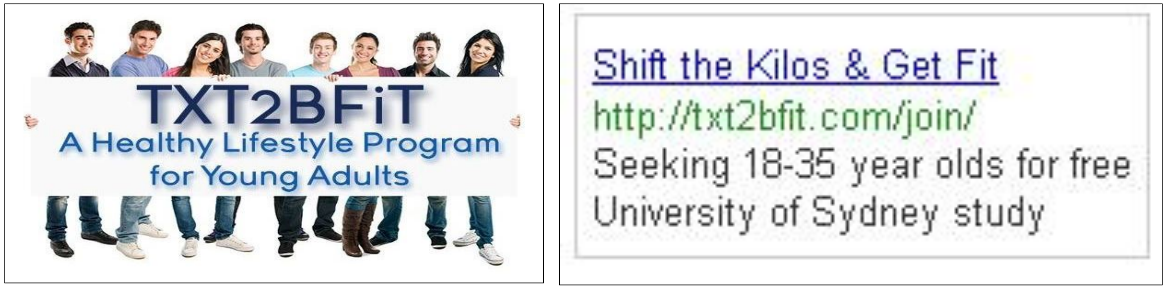

Figure 2 Example of Facebook and Google recruitment advertising

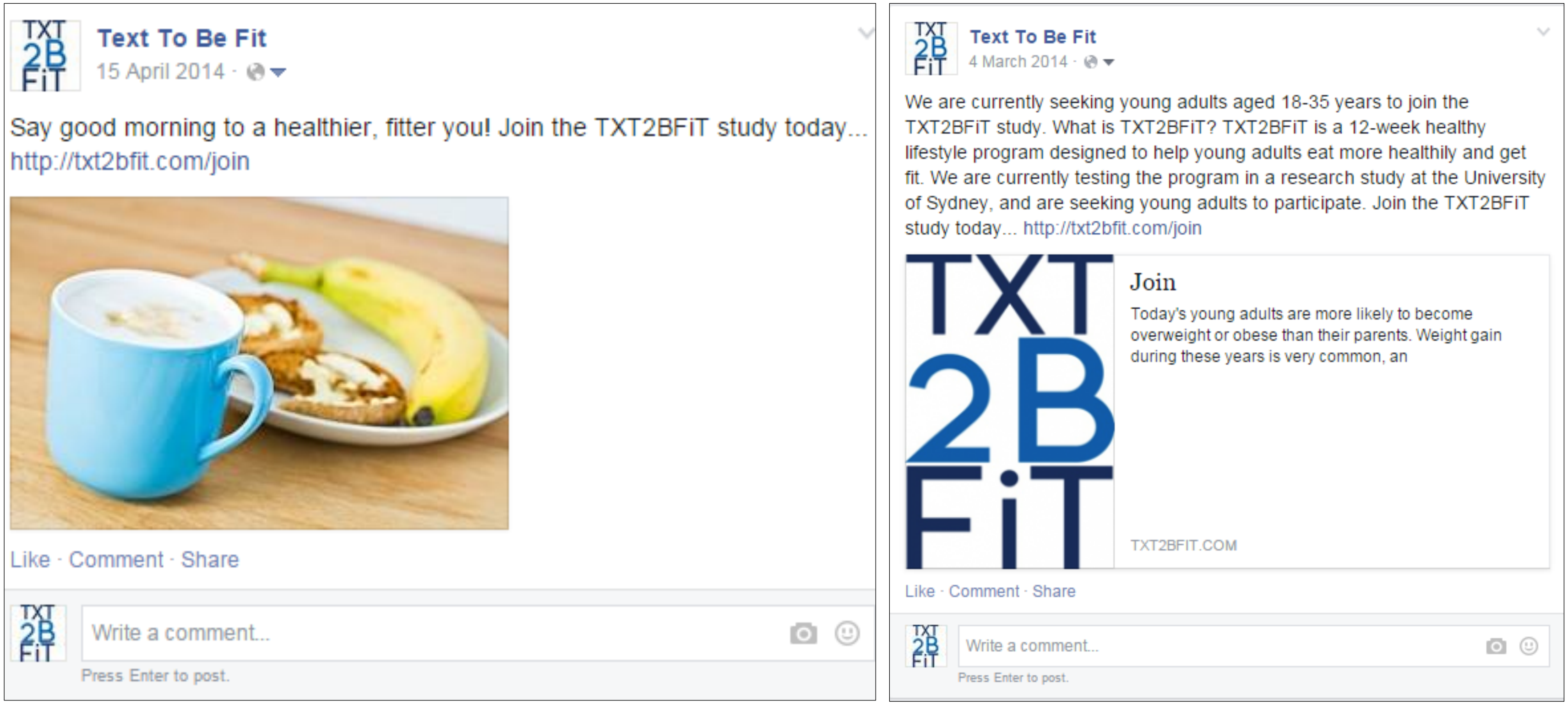

Figure 3 Example of Social Media (Facebook) recruitment advertising

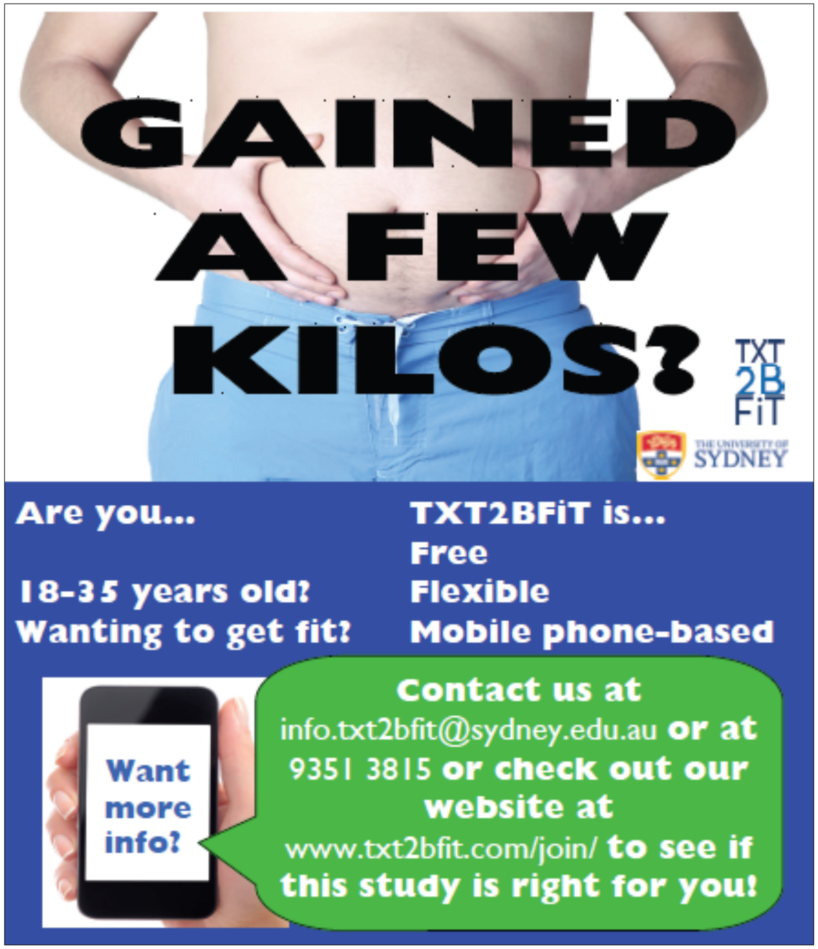

Figure 4 Example print media recruitment advertising for posters, newspaper and magazine advertisements
